# Low-Cost Microfluidic Sensors with Smart Hydrogel Patterned Arrays Using Electronic Resistive Channel Sensing for Readout

**DOI:** 10.3390/gels4040084

**Published:** 2018-10-19

**Authors:** Hsuan-Yu Leu, Navid Farhoudi, Christopher F. Reiche, Julia Körner, Swomitra Mohanty, Florian Solzbacher, Jules Magda

**Affiliations:** 1 Department of Chemical Engineering, University of Utah, Salt Lake City, UT 84112, USA; h.leu@utah.edu (H.-Y.L.); swomitra@chemeng.utah.edu (S.M.); 2 Department of Electrical & Computer Engineering, University of Utah, Salt Lake City, UT 84112, USA; navid.farhoudi@utah.edu (N.F.); christopher.reiche@utah.edu (C.F.R.); julia.koerner@utah.edu (J.K.); florian.solzbacher@utah.edu (F.S.); 3 Comprehensive Arrhythmia Research & Management Center, University of Utah, Salt Lake City, UT 84112, USA

**Keywords:** smart hydrogels, microfluidic sensors, UV photopolymerization, fast response time

## Abstract

There is a strong commercial need for inexpensive point-of-use sensors for monitoring disease biomarkers or environmental contaminants in drinking water. Point-of-use sensors that employ smart polymer hydrogels as recognition elements can be tailored to detect almost any target analyte, but often suffer from long response times. Hence, we describe here a fabrication process that can be used to manufacture low-cost point-of-use hydrogel-based microfluidics sensors with short response times. In this process, mask-templated UV photopolymerization is used to produce arrays of smart hydrogel pillars inside sub-millimeter channels located upon microfluidics devices. When these pillars contact aqueous solutions containing a target analyte, they swell or shrink, thereby changing the resistance of the microfluidic channel to ionic current flow when a small bias voltage is applied to the system. Hence resistance measurements can be used to transduce hydrogel swelling changes into electrical signals. The only instrumentation required is a simple portable potentiostat that can be operated using a smartphone or a laptop, thus making the system suitable for point of use. Rapid hydrogel response rate is achieved by fabricating arrays of smart hydrogels that have large surface area-to-volume ratios.

## 1. Introduction

A smart polymer hydrogel is a cross-linked polymer network that autonomously and reversibly swells or shrinks in response to some environment signal, such as change in the concentration of a target analyte as for example glucose [1,2]. A continuous chemical sensor can be obtained by combining a smart polymer hydrogel with a means for transducing hydrogel swelling changes into electrical signals [3]. Smart polymer hydrogels can be chemically tailored to selectively respond to many different analytes, but swelling response time is often a limiting factor for their use in sensing applications. Given that the rate of analyte mass transfer is often the rate-determining step in hydrogel response [1], it is likely that shorter response times can be achieved by fabricating smart hydrogels with large surface area-to-volume ratios. Hence, we investigate the potential use of mask-templated UV photopolymerization to produce microscopic smart hydrogel pillars with large surface area-to-volume ratios and, consequently, fast response rates. We introduce a procedure by which arrays of regularly spaced smart hydrogel pillars can be fabricated inside sub-millimeter channels located within microfluidics devices. For potential use in chemical sensing, microfluidic devices offer advantages such as potentially being low cost and requiring only small sample volumes [4]. We present sensing results obtained using arrays of regularly spaced hydrogel pillars within two different microfluidic channels, with the pillars having surface area-to-volume ratios of 40 mm^−1^ and 13.3 mm^−1^, respectively. As expected, the sensor response time is shown to decrease with an increase in surface area-to-volume ratio. The use of microscopic pillars within microfluidic sensors has been investigated in several previous studies [5,6,7]. However, here we propose a novel method for chemical sensing transduction using smart hydrogel pillars that we call resistive channel sensing. In this sensing approach, smart hydrogel pillars are fabricated within the main channel of a microfluidics device. The microchannel is then filled with phosphate buffered saline (PBS) solution to create a conductive path for ionic current. A DC voltage of less than 1.0 volts is applied to the system through the contact pads (the electrodes), which are in contact with the solution, and the induced ionic current through the channel is measured as an electrical current between the electrodes. Once the analyte reaches the smart hydrogel pillars, the pillars shrink or swell, thereby changing the resistance in the main microchannel that results in a change of the measured current. This sensing approach is similar to the well-studied technique known as microfluidic resistive pulse sensing (MRPS), in which changes in the electrical resistance of a microfluidic channel are used to determine the size of nanoparticles that pass through a microfluidic channel [8]. The principal aims of this proof-of-concept study are firstly to determine the feasibility of fabricating microscopic smart hydrogel pillar arrays with large surface area-to-volume ratios inside microfluidics channels, and secondly to determine the reduction of response time that can be attributed to the use of smart hydrogel pillars within microfluidic sensors.

## 2. Results and Discussion

### 2.1. Fabrication of Microfluidic Channels

**Figure 1:**
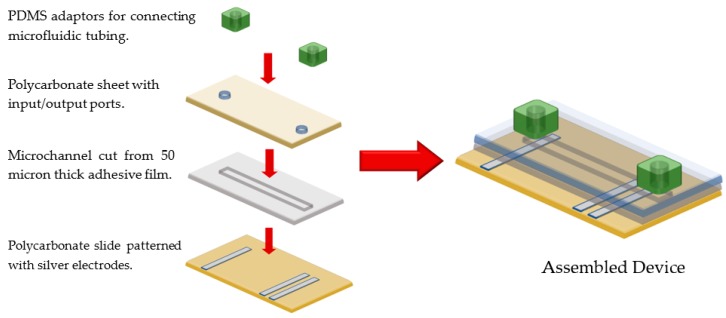
Assembly of microfluidic sensing device for in situ patterning of smart hydrogels.

The microfluidic sensing devices were synthesized using a low-cost fabrication approach with the microfluidic channels fabricated employing a computer controlled cutting plotter [9]. The sensing devices consist of 3 layers. The bottom layer in the sensing device was a rectangular piece of polycarbonate (40 mm × 75 mm × 0.25 mm) with silver paste electrodes (MG Chemical) stenciled (1 mm × 25 mm × 0.04 mm) onto the surface. The center layer was a polyvinyl chloride (PVC) adhesive film that binds the layers together and that also serves as the microchannel structure. The PVC layer has a thickness of 50 µm. The top layer was another rectangular piece of polycarbonate (25 mm × 75 mm × 0.25 mm) with holes punched through the top to access the microfluidic channels. The top layer is slightly smaller than the bottom layer to allow access to the electrodes for measurement. To make interfacing with the device simple, PDMS tubing connectors were fabricated in the lab and cored using similar methods in prior work [10]. Figure 1 shows an illustration of the assembly of the microfluidic sensing device. The channel designs were created in AutoCAD (Version: 2016; Autodesk, Inc., San Rafael, CA, USA) and then cut with the knife plotter (Model CAMEO 2; Silhouette America Inc.). The microchannel has a length of 35 mm, a width of 1.6 mm, and a depth of 50 μm.

### 2.2. UV Photopolymerization of Smart Hydrogel Pillar Arrays within Microfluidic Channels

**Figure 2:**
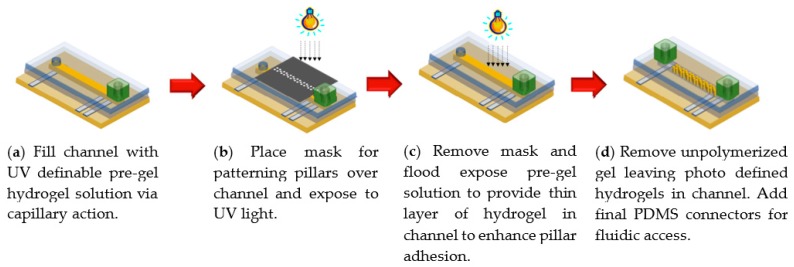
Patterning of hydrogel pillars in microfluidic channel.

The pillars were fabricated inside an enclosed microchannel using an in situ photopolymerization technique. Once the 3-layer microfluidic device was cut and assembled, the 13 wt% pre-gel hydrogel solution (see more details in Section 4), which contained 80 mol% acrylamide, 8 mol% 3-acrylamidophenylboronic acid, 10 mol% *N*-[3-(dimethylamino)propyl]methacrylamide, 2 mol% *N*,*N*′-methylenebisacrylamide and a free-radical photoinitatior was introduced into the microchannel using capillary forces. Subsequently, a dark field photomask with the desired pillar design was placed over the channel. Collimated UV light was used to polymerize the hydrogel to form pillars within the microchannel (Figure 2). After the first photo patterning was complete, the mask was removed and the entire channel containing pre-gel hydrogel solution was flood exposed to the UV light for another quarter of the previous masked exposure time. This step is very important, as it polymerizes a thin hydrogel layer across the channel to enhance adhesion of the hydrogel pillars to the channel and to keep their regular arrangement. When this step was not carried out, it was observed that the patterned pillars did not keep their locations in the channel and were easily flushed out by the surrounding flow. To vary the hydrogel surface area-to-volume ratio, we fabricated two geometrically similar square arrays. The first array has a pillar diameter (as defined by the UV mask) of 100 μm, and a spacing of 200 μm between the centers of the pillars. The pillar diameter was 300 μm in the second array, with a spacing of 600 μm between the centers of the pillars. However, the pillar height (50 μm) and the fraction of the total area occupied by the pillars (19.6%) were the same in both arrays.

### 2.3. Response of the Hydrogel Pillars to Cyclic Changes in pH

In proof-of-concept response tests, the microfluidics sensor of Figure 2 was subjected to cyclic changes in pH between 7.5 and 10.5. The hydrogel studied here contains both cationic tertiary amines and anionic phenylboronic acid moieties. However, the net hydrogel charge is negative at pH 7.5, and even more so at pH 10.5 [11]. Hence the hydrogel is expected to swell when pH is increased from 7.5 to 10.5. To make this swelling change easier to visualize with an optical microscope, we performed the pH response tests in a low ionic strength saline buffer (1/12× PBS). This reduction in salinity increases the pillar diameter at all pH values, because addition of salt causes hydrogels to shrink by reducing the environmental chemical potential value of water.

**Figure 3:**
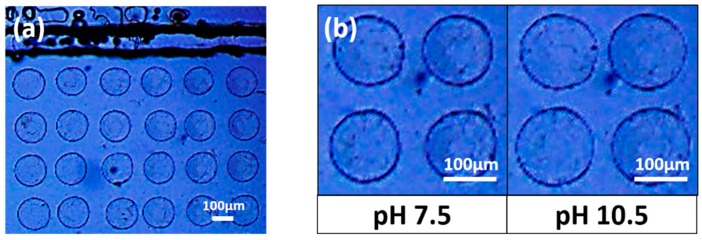
Top-down view of an array of smart hydrogel pillars fabricated by passing UV light through a mask containing an array of circular apertures of diameter 100 μm. (**a**) Smart hydrogel pillars surrounded by 1/12× PBS solution at pH 7.5 (**b**) Enlarged photograph showing the increase in pillar diameter that occurs when the pH value is increased from 7.5 to 10.5.

Figure 3a shows a micrograph of the array of smart hydrogel pillars as viewed top down in 1/12× PBS buffer at pH 7.5. This micrograph confirms that we succeeded in fabricating a regularly spaced array of smart hydrogel pillars within a microfluidics channel. Figure 3b compares the pillar diameter at pH 7.5 and 10.5. The pillars swell with increase in pH for the reasons discussed above.

**Figure 4:**
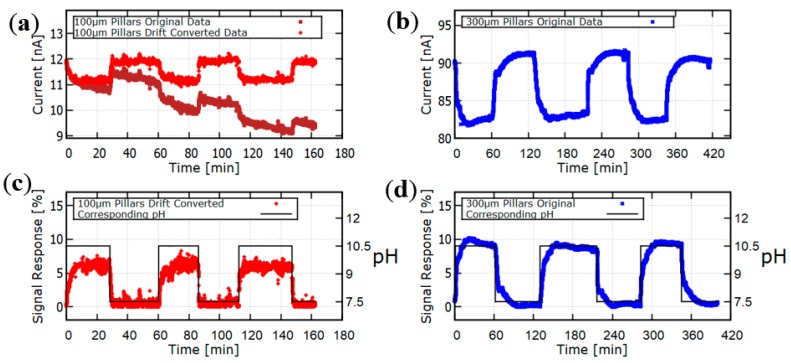
The time-dependent value of the sensor ionic current (**a**,**b**) and the Signal Response % (**c**,**d**) for periodic changes in pH between 7.5 and 10.5. The pillar diameter as defined by the UV mask was 100 μm (**a**,**c**) or 300 μm (**b**,**d**). In (**c**), the Signal Response % has been corrected for baseline drift.

When the hydrogel pillar diameter changes due to the change in pH, this changes the value of the ionic current detected by the potentiostat at fixed voltage, as shown by the results presented in Figure 4. Figure 4 shows the time-dependent behavior of the sensor current at fixed voltage as the pH value is periodically changed between 7.5 and 10.5. Results are presented for two different devices, one containing pillars of diameter 100 μm, and the other containing pillars of diameter 300 μm. At the higher pH value, the hydrogel pillars swell, which corresponds to a minimum in the value of the ionic current. The conductance of the microfluidics channel is proportional to both the ion concentration and the cross-sectional area available for current flow. Since the ionic strength was fixed at 25 mOsm/kg in these experiments, the oscillation in current observed in Figure 4 can be attributed to changes in the microfluidics channel cross-sectional area that occur as the pillars shrink and swell. Figure 4 also contains results for the time-dependent *Signal Response %*, defined as

(1) $$Signal\;Response\;\%=\frac{I_{base}-I}{I_{base}}\times 100\%$$

where *I* is the value of the ionic current at a given time *t*, and *I_base_* is the maximum current value measured at times at which the pH value equals 7.5. The value of *I_base_* was substantially different for the two devices studied (Figure 4), probably due to variabilities in the device fabrication procedure. Nonetheless, the signal response, calculated as a percentage (Equation (1)) was quite similar for the two devices studied (Figure 4). Most of the pH response of the smart hydrogel studied here occurs near pH 7, because this is close to the pK_a_ value of PBA inside polyampholytic hydrogels [11]. Hence this sensor could not be used to detect changes in pH between values less than 6, or to detect changes in pH between values greater than 8 (unless we changed the hydrogel). Based upon the results in Figure 4, we estimate that the sensor has a resolution of about 0.1 pH units near pH 7.

**Figure 5:**
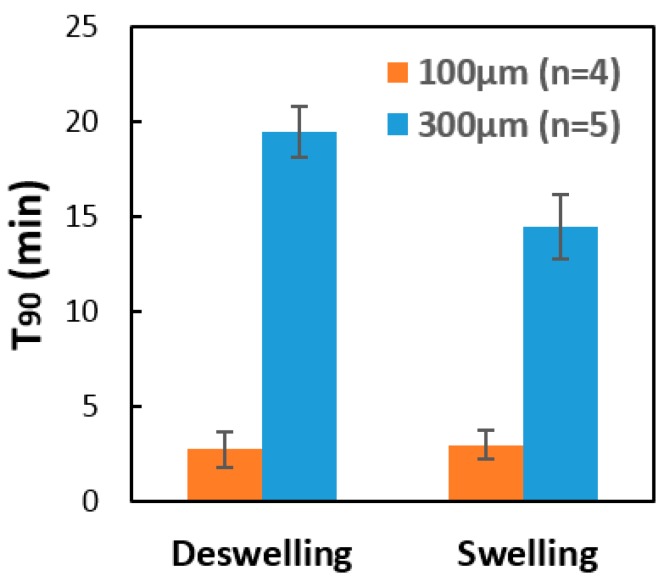
The effect of the pillar diameter upon the T_90_ response time of the microfluidic sensor, as calculated using the response data given in Figure 4. Comparison is made between the results obtained using pillars of diameter 100 μm and 300 μm, with surface area-to-volume ratios of 40 mm^−1^ and 13.3 mm^−1^, respectively. As expected, the response time is substantially smaller for the sensor that utilizes smaller diameter pillars.

The pH response data in Figure 4 was used to calculate the T_90_ response times of the two sensor devices studied. The results are presented in Figure 5. For both devices, the swelling response time is shorter than the shrinking response time. This may potentially be explained as follows. When a hydrogel starts to shrink, it shrinks first at its outer surface, thereby creating an outer surface film with a low permeability that retards further diffusion of the target analyte into the hydrogel. This, of course, tends to increase the hydrogel response time. For both swelling and shrinking response, Figure 5 shows that the response time is smaller for sensors containing smaller diameter hydrogel pillars. Comparison is made in Figure 5 between T_90_ response times obtained using pillars of diameter 100 μm and diameter 300 μm, with surface area-to-volume ratios of 40 mm^−1^ and 13.3 mm^−1^, respectively. The increase in the surface area-to-volume ratio by a factor of 3 is observed to reduce the sensor response time, averaged over both swelling and shrinking response times, by a factor of approximately 7.

## 3. Conclusions

In this work, we have demonstrated a method for fabricating low-cost and fast-responding smart hydrogel sensors inside microfluidics channels using soft material microfabrication techniques. The use of photolithographic methods to create micrometer scaled smart hydrogel structures inside a microchannel reduces the cost for this device and removes the need for cleanroom facilities. While in this work we did use a UV source from a mask aligner, a low-cost collimated UV source (such as from Omnicure Inc.) would have been sufficient to create the micropillar arrays.

In the current work, arrays of pH-responsive smart hydrogel pillars were fabricated within a microfluidics channel with surface area-to-volume ratios as large as 40 mm^−1^. The pH response of these pillars was transduced into an electrical signal using a novel technique termed *resistive channel sensing*. The electronic signal obtained using this microfluidic pH sensor was shown to be reversible and reproducible. The response time of the microfluidic pH sensor was shown to decrease with increase in the surface area-to-volume ratio of the hydrogel pillars. The fabrication process presented here is a low-cost way to solve a long-standing problem of smart hydrogel analytical devices: namely, their long response times.

## 4. Materials and Methods

The smart hydrogels studied in this work were both glucose- and pH-responsive. As discussed in reference [12], these hydrogels, which contained 13 wt% of the monomers, were copolymers containing 80 mol% acrylamide from Fisher Scientific (Hampton, NH, USA), 8 mol% 3-acrylamidophenylboronic acid from Achemo (Hong Kong, China), 10 mol% *N*-[3-(dimethylamino)propyl]methacrylamide from Polysciences Inc. (Warrington, FL, USA), and 2 mol% *N*,*N*’-methylenebisacrylamide from Sigma-Aldrich (St. Louis, MO, USA). The hydrogels were polymerized via crosslinking copolymerization [1] using lithium phenyl-2,4,6-trimethylbenzoylphosphinate from Sigma-Aldrich (St. Louis, MO, USA) as the UV free radical initiator. The light source was a collimated Hg-vapor lamp. While patterning the hydrogel pillars, a dark field chromium photomask with the desired pillars pattern was placed over the channel. Collimated UV light from a mask aligner (Model 206; OAI, San Jose, CA, USA), with an initial intensity of 13.5 W/cm^2^ and an exposure time of 5.5 s, was used to polymerize the hydrogel to form pillars within the microchannel. After the photo patterning was complete, the mask was removed. Another 1.5 s of UV exposure was flood applied to the channel itself. This process created a thin film of hydrogel between the pillars to keep the pillars from being flushed away during the introduction of analyte solutions. The UV light intensity decreased slightly from its initial value at the beginning of the experiments. Hence, the exposure time was adjusted accordingly to ensure a constant exposure dose for all experiments. A syringe pump (Model 780212; KD Scientific Inc., Holliston, MA, USA) was used to withdraw analyte solutions from one of two reservoirs (pH 7.5 and pH 10.5 in 1/12× PBS) and into the microfluidic sensors. For the sensor containing the smaller pillars, the syringe pump connection was switched between the reservoirs every 30 min, and the flow rate was 10 μL/min. For the sensor containing the larger pillars, the syringe pump connection was switched between the reservoirs every 60 min, and the flow rate was 10 μL/min. This flow rate implies a Reynolds number value of less than 100; the ionic current flow attributable to this flow is of order 1 to10 nA. The ionic current within the main microfluidics channel was measured using a potentiostat (EmStat3+) using a three-electrode configuration. One electrode pad was connected to the working electrode, while the other two were connected to the counter electrode and reference electrode pads. The system operates by applying a small bias voltage and reading the resulting current across the microchannel. The Chronoamperometry method was used to record the current data in PSTrace (Verson 5.2, Houten, The Netherlands), using a software application that came with EmStat3+. A 60 s pretreatment with a constant DV voltage of 0.3 volts was applied before data collection; the same DC voltage was then applied again throughout the entire experimental period.

The targeted solution was introduced into the microfluidic channel using the syringe pump with a flow rate of 10 μL/min for at least 20 min before imaging the pillars.

To measure the time-dependent response of the pillar diameter, a digital camera (Model LCMOS05100KPA; ToupTek, Hangzhou, China), installed on a polarizing binocular microscope (Model G508, Unico, Dayton, OH, USA), was used to take photos of the sensor pillar array every 30 s. The syringe pump was used to flow 1/12× PBS into the sensor at a flow rate of 10 μL/min, with the pH value of this solution increasing with time from 7.5 to 10.5 while photos were being taken. The photos were then analyzed using the oval tool from Image J [13] to calculate the diameter of the pillar.
